# Serum of myeloproliferative neoplasms stimulates hematopoietic stem and progenitor cells

**DOI:** 10.1371/journal.pone.0197233

**Published:** 2018-05-31

**Authors:** Richard K. Lubberich, Thomas Walenda, Tamme W. Goecke, Klaus Strathmann, Susanne Isfort, Tim H. Brümmendorf, Steffen Koschmieder, Wolfgang Wagner

**Affiliations:** 1 Helmholtz Institute for Biomedical Engineering, Stem Cell Biology and Cellular Engineering, RWTH Aachen University Medical School, Aachen, Germany; 2 Institute for Biomedical Engineering–Cell Biology, RWTH Aachen University Medical School, Aachen, Germany; 3 Department of Obstetrics and Gynecology, RWTH Aachen University Medical School, Aachen, Germany; 4 Institute for Transfusion Medicine, RWTH Aachen University Medical School, Aachen, Germany; 5 Department for Hematology, Oncology, Hemostaseology and Stem Cell Transplantation, Faculty of Medicine, RWTH Aachen University, Aachen, Germany; Hong Kong University of Science and Technology, CHINA

## Abstract

**Background:**

Myeloproliferative neoplasms (MPN)—such as polycythemia vera (PV), essential thrombocythemia (ET), and myelofibrosis (MF)—are typically diseases of the elderly caused by acquired somatic mutations. However, it is largely unknown how the malignant clone interferes with normal hematopoiesis. In this study, we analyzed if serum of MPN patients comprises soluble factors that impact on hematopoietic stem and progenitor cells (HPCs).

**Methods:**

CD34^+^ HPCs were cultured in medium supplemented with serum samples of PV, ET, or MF patients, or healthy controls. The impact on proliferation, maintenance of immature hematopoietic surface markers, and colony forming unit (CFU) potential was systematically analyzed. In addition, we compared serum of healthy young (<25 years) and elderly donors (>50 years) to determine how normal aging impacts on the hematopoiesis-supportive function of serum.

**Results:**

Serum from MF, PV and ET patients significantly increased proliferation as compared to controls. In addition, serum from MF and ET patients attenuated the loss of a primitive immunophenotype during *in vitro* culture. The CFU counts were significantly higher if HPCs were cultured with serum of MPN patients as compared to controls. Furthermore, serum of healthy young *versus* old donors did not evoke significant differences in proliferation or immunophenotype of HPCs, whereas the CFU frequency was significantly increased by serum from elderly patients.

**Conclusion:**

Our results indicate that serum derived from patients with MPN comprises activating feedback signals that stimulate the HPCs–and this stimulatory signal may result in a viscous circle that further accelerates development of the disease.

## Introduction

Myeloproliferative neoplasms (MPN) comprise a heterogeneous group of acquired clonal disorders. *BRC-ABL*-negative MPNs include the three ‘classical’ entities polycythemia vera (PV), essential thrombocythemia (ET), and primary myelofibrosis (PMF) [1]. They are characterized by excessive production of mature cells belonging to at least one myeloid cell lineage, and they are associated with clinical complications such as arterial and venous thrombosis, major hemorrhage, progressive bone marrow fibrosis, and a tendency towards leukemic transformation [2–4]. During the last decade, understanding of the pathophysiology has been greatly improved by identification of characteristic genetic aberrations, i.e. mutations in the genes encoding Janus kinase 2 (*JAK2*), calreticulin (*CALR*), and myeloproliferative leukemia virus oncogene (*MPL*). The JAK2^V617F^ mutation can be found in about 95% of PV and 50–60% of ET and PMF patients [1, 5], and the functional relevance of this cell-intrinsic modification has been extensively explored [6].

It is however largely unclear how the malignant clone interferes with the residual normal hematopoiesis. It has been suggested that the bone marrow is remodeled into a self-reinforcing leukemic niche [7]. Moreover, cytokine associated effects play a crucial role in pathogenesis of MPN. For example, transforming growth factor beta 1 (TGF-ß1) is involved in bone marrow fibrosis [8]. Cytokines may also support dominance of the malignant clone: interleukin-33 enhances cytokine production and colony formation of CD34+ MPN-HPCs [9]. Furthermore, tumor necrosis factor alpha (TNF-α) and lipocalin-2, have been identified to actively suppress residual normal hematopoiesis and facilitate clonal expansion of JAK2^V617F^ cells [10, 11]. Thus, paracrine and systemic mechanisms seem to contribute to development of MPN [12].

We have previously demonstrated that serum taken from patients after hematopoietic stem cell transplantation enhances proliferation and maintenance a primitive immunophenotype of HSCs *in vitro* [13]. These results indicated that serum comprises soluble factors that recruit the quiescent stem cells into cycle when necessary. Furthermore, we demonstrated that serum of patients with myelodysplastic syndromes (MDS) contains soluble factors that enhance proliferation of normal HPC as a feedback mechanism to rescue physiologic blood formation [14]. However, it was yet unknown if these activating feedback loops are primarily triggered by low cell counts, or if they are also activated in diseases with higher cell numbers and aberrant hematopoiesis such as MPN. A better understanding of these feedback mechanisms may help to understand how normal hematopoiesis is influenced by the malignant MPN clone–and it may provide new targets for therapeutic interventions.

## Material and methods

### Serum samples of MPN patients and healthy donors

Patients with MF, ET, and PV were diagnosed according to the guidelines of the European Society for Medical Oncology and 1 ml of serum was provided by the Department for Hematology, Oncology, Hemostaseology and Stem Cell Transplantation. Additional clinical information is provided in S1 Table. Blood samples from healthy control groups were collected by the Institute of Transfusion Medicine (S2 and S3 Tables). All samples were taken after informed and written consent and the Ethical Committee of RWTH Aachen Medical School has specifically approved this study (permit numbers: EK 155/09, EK127/12, and EK 041/15, respectively). For separation of serum fresh blood samples were incubated at room temperature for 1 h to allow coagulation and subsequently centrifuged at 2,000 g for 10 min. Supernatant was harvested and stored at -80°C.

### Culture of hematopoietic stem and progenitor cells

Cord blood (CB) was obtained from the Department of Obstetrics and Gynecology after informed and written consent of the mothers according to the guidelines of Ethics Committee of RWTH Aachen Medical School and the Ethics Committee of RWTH Aachen Medical School has specifically approved this study (permit number: EK187/08). Mononuclear cell enrichment was performed by density gradient centrifugation with Biocoll (Biochrom GmbH, Berlin, Germany) and CD34^+^ HPCs were enriched by a CD34 MicroBead Kit (Miltenyi BioTec, Bergisch Gladbach, Germany). Cells were cultured in 24 well plates with 450 μl StemSpan serum free expansion medium (Stem Cell Technologies, Vancouver, Canada) supplemented with 10 ng/ml stem cell factor (SCF; PeproTech GmbH, Hamburg, Germany), 20 ng/ml thrombopoietin (TPO; PeproTech), 10 ng/ml fibroblast growth factor 1 (FGF-1; PeproTech) and 10 mg/ml heparin (Rotexmedica, Trittau, Germany). Furthermore, each well was supplemented with 10% (50 μl) serum from either MPN patients or control group to directly compare the effect of all serum samples in parallel. Each experiment was performed with individual cord blood samples and for inter-experiment comparison we normalized the data by the means of corresponding healthy control measurements.

### Analysis of cell division history

Cell division history was monitored by retention of carboxyfluorescein diacetate N-succinimidyl ester (CFSE; Sigma Aldrich, Steinheim, Germany) [15]. In brief, CD34^+^ cells were labeled in a 3 μM CFSE solution in PBS with 0.1% fetal calf serum (FCS) for 10 min at 37°C in the dark. Staining was interrupted by adding 5 ml of cold PBS with 10% FCS and incubation on ice for 5 min followed by two washing steps and flow cytometric analysis, as indicated below.

### Immunophenotypic analysis

Surface marker expression and residual CFSE staining was analyzed after five days of culture. The cells were washed in PBS and stained with CD34-allophycocyanin (APC Mouse Anti-Human CD34, clone 581; Becton Dickinson [BD], Franklin Lakes, NJ, USA), CD133-phycoerythrin (CD133/2 (AC141)-PE, clone AC141; Miltenyi Biotec, Bergisch Gladbach) and CD45-V500 (V500 Mouse Anti-Human CD45, clone HI30, BD). To discriminate dead cells we used 7-Aminoactinomycin D (7-AAD Viability Staining Solution; BioLegend, San Diego, USA) or propidium iodide (Propidium Iodide Staining Solution, BD) staining shortly before measurement. Flow cytometric measurements were performed with a FACS Canto II flow cytometer (BD) and data were analyzed using Flowjo software V10 (FlowJo, LLC, Ashland, USA).

### Colony forming unit assays

Colony forming unit (CFU) potential was determined as described before [16]. In brief, 20,000 HPCs were cultured as described above for seven days and their progeny was subsequently re-seeded in different dilutions (1:10, 1:100 and 1:1000) in 24-well culture conditions with 500 μl methylcellulose media per well (HSC-CFU lite with EPO; Miltenyi Biotec). After 14 days colonies were counted and classified according to the manufacturer’s instructions. The differences in CFU frequency might be due to effects on maintenance and/or proliferation.

### Statistics

To estimate the probability of differences we have applied the two-sided Student’s T-test unless otherwise specified. Probability values of p < 0.05 were considered statistically significant. All bar charts indicate mean ± standard error of the mean (SEM), if not otherwise stated. Association of clinical parameters and functional affection on HPCs was analyzed using Pearson correlation in Microsoft Excel and Linear regression analysis using GraphPad Prism version 6.

## Results

### Serum of patients with myelofibrosis stimulates hematopoietic stem and progenitor cells

**Fig 1:**
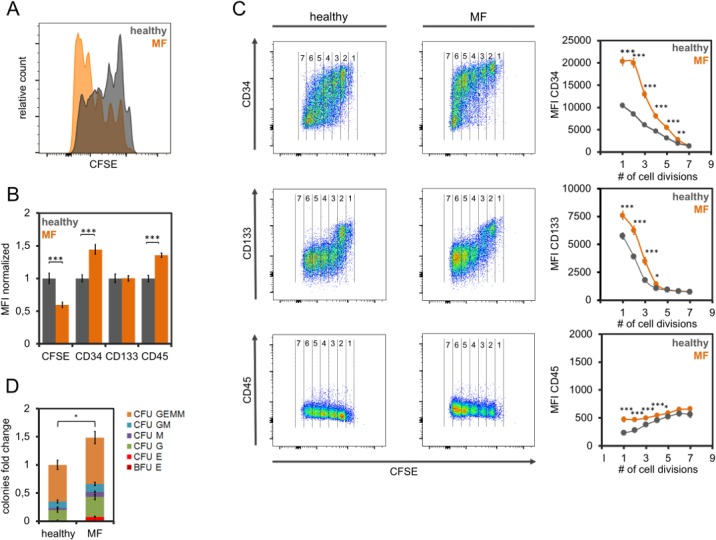
Effects of serum from myelofibrosis patients on hematopoietic stem and progenitor cells. (A) Exemplary histogram to demonstrate higher proliferation of CD34^+^ HPCs in culture medium supplemented with serum of MF patients as compared to serum from healthy controls. After five days, residual staining of carboxyfluorescein succinimidyl ester (CFSE) is lower with MF-serum. Each peak corresponds to one cell division. (B) Mean fluorescence intensity (MFI) of flow cytometric measurements of HPCs that were cultured for 5 days in parallel with 12 MF and 15 control samples. Values were normalized by the mean MFI of healthy controls. (C) Immunophenotypic analysis in relation to the number of cell divisions (according the residual CFSE staining). The numbers provide estimates for cell divisions. (D) CD34^+^ cells were cultured for seven days in parallel with serum-supplements of MF patients (n = 9) or controls (n = 12) and the number of colony forming units (CFUs) was then analyzed. * p<0.05, ** p<0.01, ***p<0.001.

Initially, we analyzed if serum of primary, post-PV or post-ET MF affects proliferation of HPCs. To this end, we used 12 serum samples from MF patients and 15 samples from healthy controls to supplement culture media of CD34^+^ cells in parallel. After five days, proliferation was estimated by residual CFSE staining (Fig 1A). In serum of MF patients, HPCs exhibited significantly reduced CFSE intensity as compared to controls, indicating that proliferation was accelerated (Fig 1B; p < 0.001). The same growth promoting effect was also observed with two additional independent CB samples.

Increased proliferation of HPCs during *in vitro* culture is usually associated with a decline of immunophenotypic markers for stem and progenitor cells, such as CD34 and CD133 [17–21]. Notably, the mean overall expression of CD34 was higher for HPCs cultivated with MF serum as compared to controls (Fig 1B). On the other hand, MF serum resulted in a moderate upregulation of CD45. Combined analysis with residual CFSE stain supported the notion that after the same number of cell divisions, CD34 and CD133 expression was higher if HPCs were cultured in MF-serum as compared to healthy control serum (Fig 1C). The same tendencies of immunophenotypic changes were also observed with two additional independent CB samples.

Subsequently, we analyzed if the presence of MF serum or control serum affects the maintenance and/or proliferation of cells with colony forming unit (CFU) potential. For this purpose, HPCs were cultured with medium supplemented with each of the 12 MF or the 15 samples of the control group for seven days. The progeny was then covered in methylcellulose for 14 days and the colony frequency was determined. Serum of MF patients resulted in a 1.5-fold increase of CFU-numbers (p = 0.015), whereas no lineage-specific shift was observed (Fig 1D).

### Serum of patients with ET has similar hematopoiesis-supportive effects

**Fig 2:**
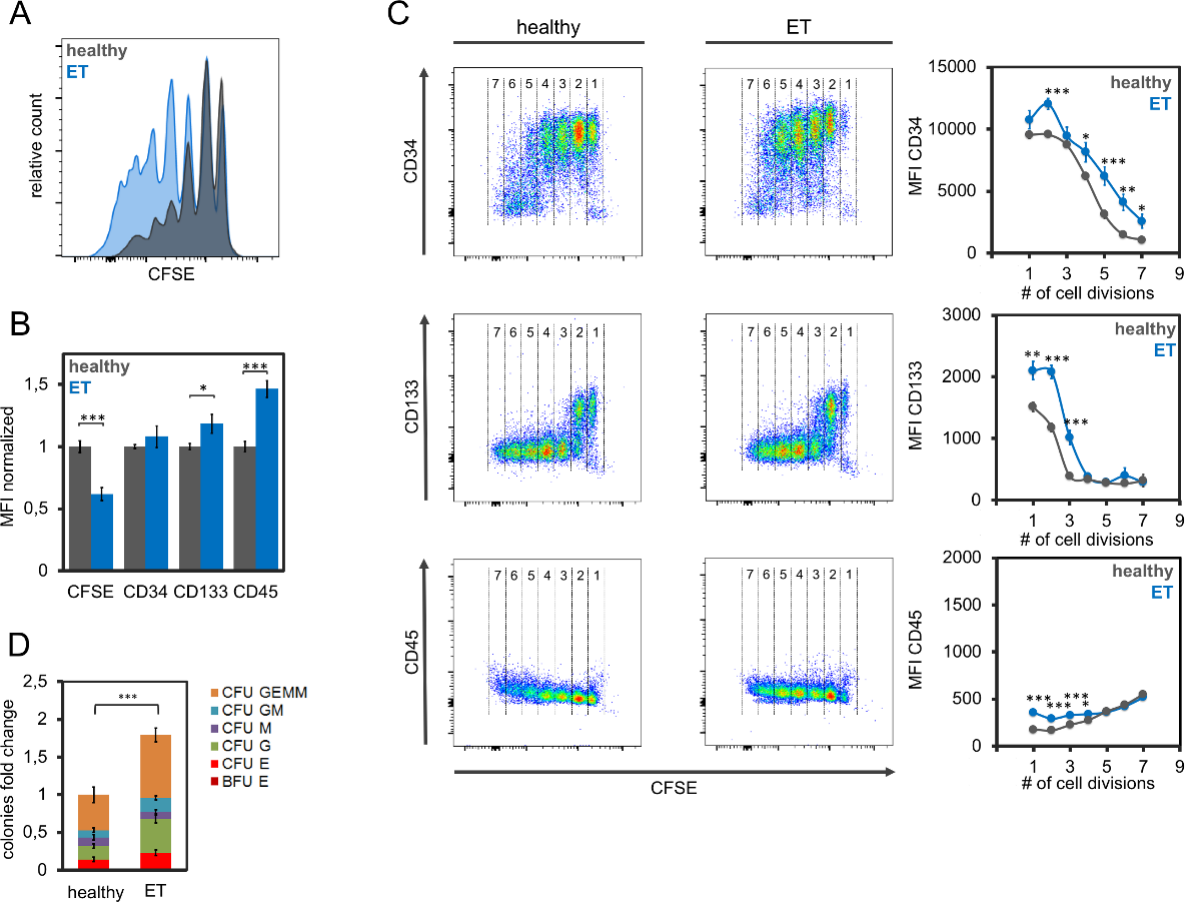
Effects of serum from essential thrombocythemia patients on hematopoietic stem and progenitor cells. (A) Hematopoietic stem and progenitor cells proliferate faster if cultured with serum of ET-patients as compared to healthy controls. Residual CFSE staining was analyzed after five days. (B) Mean fluorescence intensity (MFI) of flow cytometric measurements of HPCs that were cultured for 5 days in parallel with 15 ET and 15 control samples (normalized to controls). (C) Immunophenotypic analysis in relation to the number of cell divisions (according the residual CFSE staining). The numbers provide estimates cell divisions. (D) Cell culture with serum of ET patients (n = 15) resulted in higher colony forming unit (CFU) frequency than serum of healthy controls (n = 15). * p<0.05, ** p<0.01, ***p<0.001.

**Fig 3:**
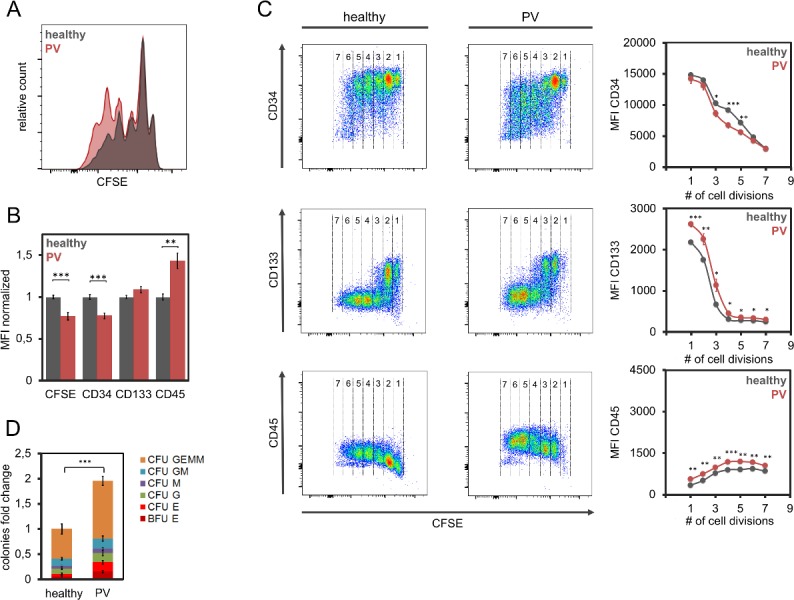
Effects of serum from polycythemia vera patients on hematopoietic stem and progenitor cells. (A) Residual CFSE staining after five days indicated that serum of PV patients had only a moderate effect on proliferation of CD34^+^ cells as compared to serum of healthy donors. (B) Mean fluorescence intensity (MFI) of flow cytometric measurements of HPCs that were cultured for 5 days in parallel with 8 PV and 15 control samples (normalized to controls). (C) Immunophenotypic analysis in relation to the number of cell divisions (according the residual CFSE staining). The numbers provide estimates for the number of cell divisions. (D) Cell culture with serum of PV patients (n = 8) resulted in higher colony forming unit (CFU) frequency than serum of healthy controls (n = 15). * p<0.05, ** p<0.01, ***p<0.001.

Essential thrombocytosis is characterized by the overproduction of platelets by megakaryocytes. We used serum of 15 ET patients and of the aforementioned 15 healthy controls to compare effects on HPCs. ET-derived serum significantly enhanced proliferation of HPCs (p < 0.001; Fig 2A). Furthermore, CD133 surface levels were significantly higher in HPCs that were cultured with ET than control serum (p < 0.05; Fig 2B). CD34 surface levels were also higher in HPCs that were cultured with ET than control serum, but this analysis did not reach statistical significance. In contrast to MF-serum the upregulation of CD34 was particularly observed in the rapidly proliferating cells (Fig 2C). CFU numbers were 1.8-fold higher when HPCs were cultured with ET-derived than control serum (p < 0.01, Fig 3D). Taken together, addition of ET-derived serum had similar growth promoting effects on HPCs as MF-derived serum.

### Serum of patients with PV has less pronounced effects on proliferation and immunophenotype

It has been demonstrated that the cytokine profile differs significantly in serum of PV and MF patients [22]. Therefore, we hypothesized that serum of these entities will have different effects on HPCs. We expanded HPCs in the presence of serum from 8 PV patients and 15 healthy controls. Proliferation was increased with PV-derived serum, albeit less pronounced than MF or ET (Fig 3A and S1 Fig). Notably, PV-derived serum did not support maintenance of primitive surface markers: expression of CD34 was moderately reduced, while mean fluorescence intensity of CD133 was not affected (Fig 3 Band 3C). Thus, PV-derived serum has an opposite effect on CD34 than MF- and ET-derived serum. Nevertheless, culture with PV-derived serum significantly increased the number of CFU 1.9-fold as compared to control serum (p<0.01) and there was no bias for specific lineages (Fig 3D).

### Correlation of hematopoiesis-supportive potential of serum with blood counts in MPN patients

**Fig 4:**
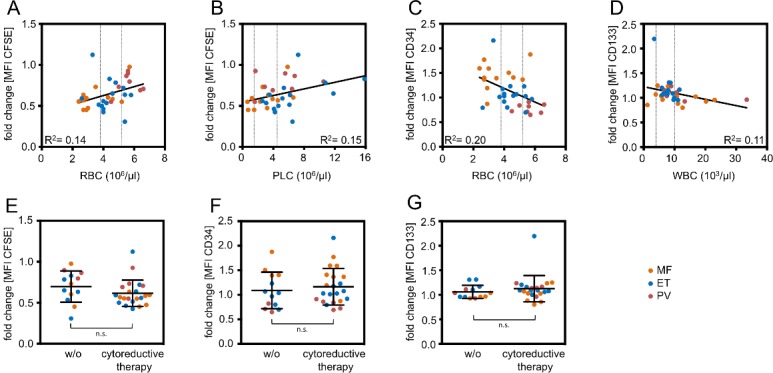
Moderate association of stimulatory effects of serum with corresponding blood counts. Mean fluorescence intensities (MFI) were normalized to healthy controls and fold changes were correlated with clinical parameters. (A) Serum taken from patients with low red blood cell (RBC) counts had in tendency higher stimulatory effect on proliferation of HPCs. MFI of residual CFSE staining was normalized by the mean of the healthy control. (B) Proliferation of HPCs was higher in serum with lower platelet counts (PLC). (C) Serum of patients with low RBC counts supported maintenance of CD34 expression better than serum of patients with high RBC counts. (D) White blood counts (WBC) revealed moderate anti-correlation with maintenance of CD133 expression in HPCs upon culture with corresponding serum samples. (E-G) Cytoreductive therapy did not impact on the stimulatory effect of patient serum with regard to (E) proliferation, (F) CD34 expression, and (G) CD133 expression (mean ± standard deviation; n.s. = not significant).

Systemic regulatory feedback signals might be directly related to clinical parameters or therapeutic regimen [23]. This analysis was performed across all the different types of MPN due to the relatively small sample numbers within each entity, but there are notoriously differences between the different MPN entities. Activation of proliferation was particularly observed in serum from patients with low red blood cell (RBC) counts and low platelet counts (PLC; Fig 4A and 4B), whereas expression of CD34 or CD133 was rather linked to RBCs or reduced white blood cell counts (WBC), respectively (Fig 4C and 4D). Furthermore, there was a moderate negative correlation of CD34 expression with hemoglobin levels, while there was no association with lactate dehydrogenase levels (S2 Fig). Some of our serum samples were taken from patients undergoing cytoreductive therapy and therefore we determined if this might impact on our results. Comparison of serum with and without cytoreductive therapy did not reveal significant differences in CFSE intensity–it was even slightly lower in patients with cytoreductive therapy (Fig 4E). Expression of CD34 and CD133 did not significantly differ between these groups (Fig 4F and 4G). Taken together, there was only a moderate association of cell counts in peripheral blood with the hematopoiesis supportive potential of MPN serum.

### Serum of elderly donors increases colony forming unit frequency

**Fig 5:**
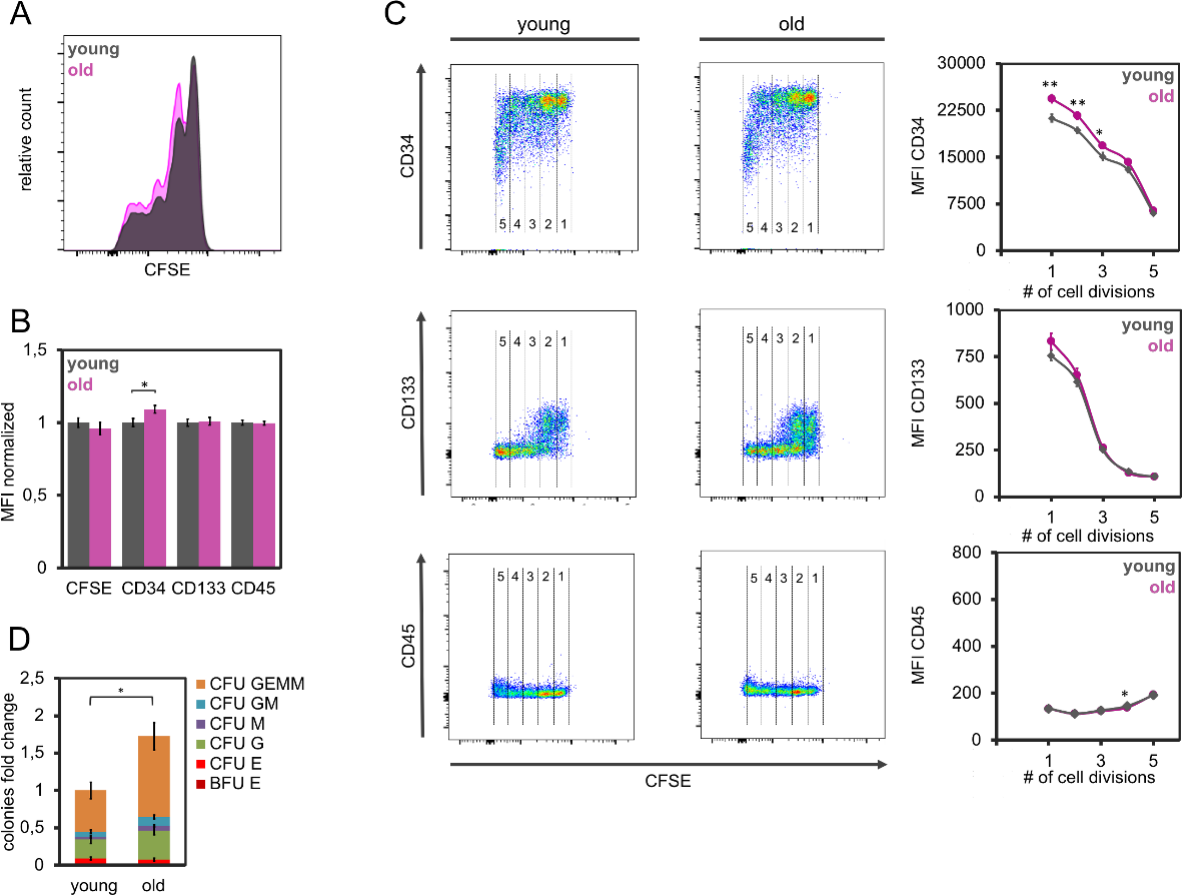
Serum of elderly donors stimulates maintenance of CFU-frequency. (A) Serum of young and old healthy donors had similar effect on proliferation of hematopoietic stem and progenitor cells (exemplary histogram, residual CFSE was analyzed after five days). (B) Mean fluorescence intensity (MFI) of flow cytometric measurements of HPCs that were cultured for 5 days in parallel with serum of 14 young (< 25 years) and 15 elderly (>50 years) donors (normalized to young donors). (C) Immunophenotypic analysis in relation to the number of cell divisions (according the residual CFSE staining). The numbers provide estimates for the number of cell divisions. (D) Cell culture with serum of elderly donors (n = 15) resulted in higher colony forming unit (CFU) frequency than serum of younger donors (n = 14). * p<0.05, ** p<0.01, ***p<0.001.

Myeloproliferative neoplasms typically occur in elderly patients. The regenerative potential of the hematopoietic system declines upon aging and therefore we reasoned that healthy aging might be encountered by similar systemic feedback mechanisms on HPCs. To address this question, we compared serum from 14 young (<25 years) and 15 elderly donors (>50 years). We did not observe significant differences in proliferation of HPC (Fig 5A), and there was only a moderate increase of CD34 expression with serum of older donors, particularly in the slow dividing fraction (Fig 5B and 5C). Notably, the CFU frequency was 1.7-fold higher if HPCs were cultured with serum of elderly donors as compared to younger donors (Fig 5D), indicating that hematopoiesis supportive feedback signals are rather enhanced upon aging.

## Discussion

Serum of MPN patients comprises soluble factors that activate non-malignant HPCs. This is in line with our previous work, where we analyzed hematopoietic reconstitution after chemotherapy: activation of the stem cell pool upon chemotherapy seems to be mediated by systemic feedback mechanisms [24] and a mathematical model suggested that this is particularly mediated by increased self-renewal rates of HPCs [25]. Furthermore, we observed similar effects with serum of MDS patients that–in contrast to MPN–is characterized by dysplasia and failure to produce functional mature blood cells [14]. Our current work indicates that regulatory loops are not exclusively dependent on cell numbers in peripheral blood, but rather on the function of mature cells.

In this study, proliferation was estimated by CFSE staining, which enables immunophenotypic analysis in relation cell division numbers. This is important as CD34 and CD133 expression declines particularly in faster proliferating cells [17–21]–but this method does not provide absolute cell numbers. Furthermore, it needs to be taken into account that the CFU frequency reflects maintenance as well as self-renewal of primitive progenitor cells. Therefore, alternative functional studies should be considered in the future to better pinpoint effects of patient serum on stem cell function, such as kinetics during culture expansion or murine transplantation models.

Many previous studies have identified aberrantly expressed cytokines in MPN and investigated their contribution to pathogenesis [22, 26–30]. Cytokine profiles differ between MPN entities. For example, PV patients exhibit higher levels of interleukin 4 (IL-4), IL-8 and granulocyte-macrophage colony-stimulating factor (GM-CSF) as compared to ET patients [22, 28]. The disease-specific cytokine patterns may explain the attenuated effects of PV serum as compared to ET and MF serum. On the other hand, various micro RNAs (miRNAs), such as iR-433, have been described as a negative regulator of proliferation and differentiation of CD34^+^ cells in MPN [31, 32]–and they may exert paracrine effects either as free circulating miRNAs or via exosomes. Overall, it appears to be likely that hematopoiesis is not mediated by a single factor, but rather by a complex combination of different stimuli that act in concert [24].

Up-regulation of hematopoiesis-supportive signals in MPN appears to be counter-intuitive since MPN is characterized by overproduction of mature blood cells. In fact, it has been suggested that normal hematopoiesis is suppressed in MPN by distinct cytokines: lipocalin-2, tumor necrose factor (TNF) β, and TNF-α are aberrantly expressed in MPN and rather interfere with normal hematopoiesis than with the malignant clone [10, 11, 33]. It is generally anticipated that the malignant MPN clone directly remodels the bone marrow niche into a self-reinforcing leukemic niche that suppresses residual hematopoiesis [7]. In this regard, the results of this study were unexpected: we demonstrate that MPN serum supports proliferation as well as maintenance of primitive immunophenotye and CFU-potential also in non-malignant HPCs. In the future, it will be important to better understand the regulatory mechanisms and how they act on normal and malignant subpopulations.

Aged HPC exhibit several functional defects under conditions of stress and regeneration [34]. On the other hand, it has been demonstrated that the number of HPCs in the bone marrow increases at least two-fold upon ageing [35]. Our results suggest that increased HPC frequency might result from activated systemic feedback signals upon aging. Serum of elderly donors gave rise to significantly higher CFU-frequency than serum of younger donors, while proliferation and immunophenotype were not affected. Hence, self-renewal of primitive HPCs might be specifically activated by soluble factors in serum of elderly donors, but this deserves further functional analysis in the future. In fact, it has been demonstrated that aged bone marrow is characterized by low-level inflammation with an increase of corresponding cytokines, such as IL-6 and TNF-α [36]. While for the age range of our healthy controls was similar for serum samples for ET and PV, the patients with MF were in average 18 years older than the controls. Hence, higher CFU-frequency with serum of MF patients might be partly attributed to higher age. On the other hand, serum of elderly healthy donors did not support proliferation of maintenance of a primitive immunophenotype of HPCs. These results indicate that there are regulatory mechanisms that compensate the declining hematopoietic potential upon aging–but the signals are not identical to the feedback loops in MPN.

## Conclusion

Serum of MPN patients comprises soluble factors that stimulate proliferation and maintain a primitive immunophenotype of normal HPCs *in vitro*. The precise nature of these feedback signals is yet unclear—presumably cytokines and other factors are involved in the regulatory process. The complex interplay of activating and deactivating factors may even shift the balance in favor of the malignant clone. Ineffective normal hematopoiesis might then contribute to a vicious circle that further contributes to activation of the malignant clone and hence disease development.

## Supporting information

S1 Fig Comparison of growth promoting effect of serum from PV, ET and MF serum. Stimulation of proliferation of HPCs by sera from polycythemia vera (PV), essential thrombocythemia (ET), and myelofibrosis (MF). Mean fluorescence intensities (MFI) of CFSE staining were normalized to corresponding measurements of healthy controls. Statistical significance was estimated by Kruskal- Wallis and Dunn's multiple comparisons test (* p<0.05). Bars indicate standard error of the mean (SEM).
(PDF)

S2 Fig Association of the stimulatory effect with hemoglobin and lactate dehydrogenase. Hemoglobin (Hb; A-C) and lactate dehydrogenase levels in blood (LDH; D-F) were compared with the stimulatory effect of patient serum on HPCs. (A,D) Proliferation was estimated by mean fluorescence intensities (MFI) of CFSE staining (normalized to healthy controls) and did not correlate with Hb or LDH. (B,E) CD34 expression (normalized to healthy controls) revealed moderate anti-correlation with Hb, which is in line with the association in red blood cell count (Fig 4C), while LDH had no clear association. (C,F) CD133 expression did not correlate with Hb or LDH.
(PDF)

S1 Table Sample information: MPN samples. (PDF)

S2 Table Sample information: Control samples from healthy blood donors. (PDF)

S3 Table Sample information: Young and old healthy blood donors. (PDF)
