# Supplementary material for: Serum of myeloproliferative neoplasms stimulates hematopoietic stem and progenitor cells
Source: PLoS One. 2018 May 31;13(5):e0197233. doi: 10.1371/journal.pone.0197233 (PMC5979002; doi:10.1371/journal.pone.0197233)
Supplement: S1 Fig — Stimulation of proliferation of HPCs by sera from polycythemia vera (PV), essential thrombocythemia (ET), and myelofibrosis (MF). Mean fluorescence intensities (MFI) of CFSE staining were normalized to corresponding measurements of healthy controls. Statistical significance was estimated by Kruskal- Wallis and Dunn's multiple comparisons test (* p<0.05). Bars indicate standard error of the mean (SEM). (PDF) [file pone.0197233.s001.pdf]

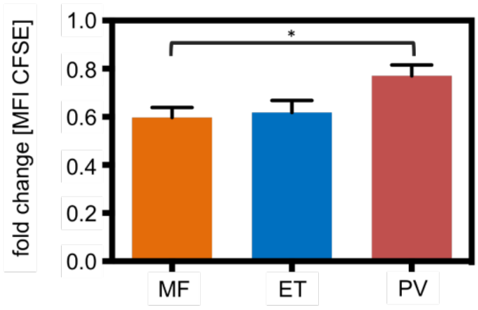

Fig S1. Comparison of growth promoting effect of serum from PV, ET and MF serum.
Stimulation of proliferation of HPCs by sera from polycythemia vera (PV), essential thrombocythemia (ET), and myelofibrosis (MF). Mean fluorescence intensities (MFI) of CFSE staining were normalized to corresponding measurements of healthy controls. Statistical significance was estimated by Kruskal-Wallis and Dunn's multiple comparisons test (* $p<0.05$). Bars indicate standard error of the mean (SEM).
