# Supplementary material for: Serum of myeloproliferative neoplasms stimulates hematopoietic stem and progenitor cells
Source: PLoS One. 2018 May 31;13(5):e0197233. doi: 10.1371/journal.pone.0197233 (PMC5979002; doi:10.1371/journal.pone.0197233)
Supplement: S2 Fig — Hemoglobin (Hb; A-C) and lactate dehydrogenase levels in blood (LDH; D-F) were compared with the stimulatory effect of patient serum on HPCs. (A,D) Proliferation was estimated by mean fluorescence intensities (MFI) of CFSE staining (normalized to healthy controls) and did not correlate with Hb or LDH. (B,E) CD34 expression (normalized to healthy controls) revealed moderate anti-correlation with Hb, which is in line with the association in red blood cell count (Fig 4C), while LDH had no clear association. (C,F) CD133 expression did not correlate with Hb or LDH. (PDF) [file pone.0197233.s002.pdf]

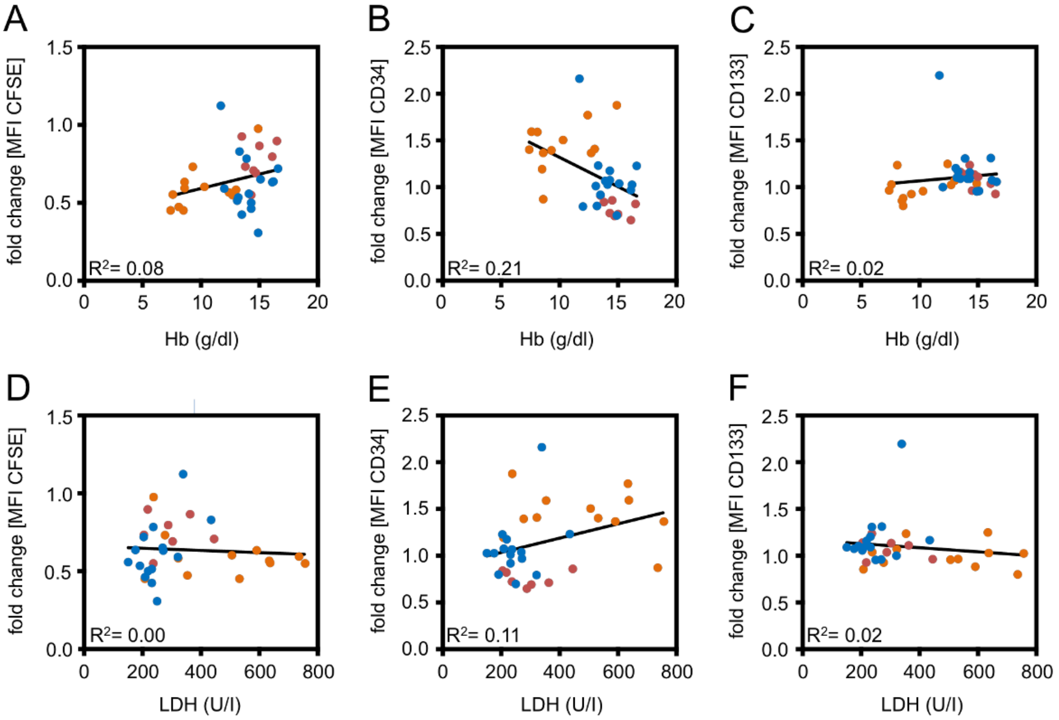

Fig S2. Association of the stimulatory effect with hemoglobin and lactate dehydrogenase. Hemoglobin (Hb; A-C) and lactate dehydrogenase levels in blood (LDH; D-F) were compared with the stimulatory effect of patient serum on HPCs. (A,D) Proliferation was estimated by mean fluorescence intensities (MFI) of CFSE staining (normalized to healthy controls) and did not correlate with Hb or LDH. (B,E) CD34 expression (normalized to healthy controls) revealed moderate anti-correlation with Hb, which is in line with the association in red blood cell count (Figure 4C), while LDH had no clear association. (C,F) CD133 expression did not correlate with Hb or LDH.
