# Supplementary material for: Serum of myeloproliferative neoplasms stimulates hematopoietic stem and progenitor cells
Source: PLoS One. 2018 May 31;13(5):e0197233. doi: 10.1371/journal.pone.0197233 (PMC5979002; doi:10.1371/journal.pone.0197233)
Supplement: S1 Table — (PDF) [file pone.0197233.s003.pdf]

S1 Table: Sample information: MPN samples

Sample	Sex	Age	RBC 10^6 / µl	WBC 10^3 / µl	Hb g/dl	Hct 100 %	PLC 10^3 / µl	LDH U/l	Epo U/l	Cytoreductive agents
MF1	m	76	2.4	10.7	7.6	0.22	231	637	432	Ruxolitinib
MF2[#]	m	65	5.2	16.8	12.7	0.43	64	757	19.5	HU, Everolimus
MF3	m	73	2.9	12.6	8.6	0.26	122	736	N/A	Ruxolitinib
MF4	m	75	2.7	11.1	8.6	0.25	334	591	N/A	Ruxolitinib
MF5	f	72	4.3	23	10.3	0.33	660	506	N/A	-
MF6[*]	m	81	2.9	1.2	8.5	0.25	73	208	N/A	Pomalidomide
MF7[#]	m	67	3	7.9	8.1	0.26	320	354	121	Anagrelide
MF8	m	78	2.7	6.1	7.4	0.24	151	532	11.3	-
MF9	m	51	5.7	5.7	14.9	0.46	586	238	N/A	-
MF10[*]	f	57	3.5	20.2	9.3	0.32	262	277	16.1	-
MF11	m	60	4.6	3.9	13	0.4	391	322	31.5	Ruxolitinib
MF12	f	75	2.7	4.5	12.4	0.35	459	634	40.5	HU
ET 1	m	55	5.3	6	16.6	0.49	537	204	N/A	HU
ET 2	m	79	4	9.5	14.3	0.41	409	209	11.8	HU
ET 3	m	37	2.6	9.9	12	0.34	519	321	N/A	HU
ET 4	f	74	3.3	3.4	11.7	0.36	723	339	32	HU
ET 5	f	37	5.8	8.2	16.1	0.47	618	271	3.2	-
ET 6	m	28	5.4	7.7	16.2	0.47	350	176	14.9	Interferon
ET 7	f	55	4	6.5	14.1	0.4	259	151	28.5	HU
ET 8	f	43	4.4	11.6	13.3	0.41	1585	435	12.6	-
ET 9	f	45	4.2	7.1	13.2	0.4	308	191	N/A	-
ET 10	f	29	3.7	5.9	13.1	0.4	610	232	11.6	HU
ET 11	m	52	4.6	9.7	15.1	0.42	1181	269	9.6	-
ET 12	f	34	4.8	8.7	13.9	0.4	1076	237	13.1	-
ET 13	m	51	4	9.4	13.5	0.42	422	232	N/A	HU
ET 14	f	64	5.4	11.1	14.9	0.46	649	250	3.8	-
ET 15	f	79	3.9	7.9	14.3	0.41	466	219	12.2	HU
PV 1	f	42	5.7	7.5	16.1	0.5	1053	288	1.7	-
PV 2	m	40	5.6	13.3	16.5	0.49	555	218	4.2	-
PV 3	m	47	6.6	33.4	14.5	0.5	643	445	4.3	HU
PV 4	f	50	5.6	6.5	13.5	0.43	168		2.1	HU
PV 5	f	44	5.5	10.7	15	0.55	678	363	0.9	-
PV 6	f	72	6.4	8.1	14.7	0.48	393	303	N/A	Anagrelide
PV 7	m	62	5	8.1	13.8	0.45	289	205	5.7	HU
PV 8	f	63	4.5	8.9	14.3	0.44	159	237	N/A	HU

RBC = red blood cell count; WBC = white blood cell count; Hb = hemoglobin; Hct = hematocrit; PLC = platelet count; LDH = lactate dehydrogenase; EPO = erythropoietin; HU = hydroxyurea; [#,*] = samples were taken from the same patient but at different time points.
