# Supplementary material for: Serum of myeloproliferative neoplasms stimulates hematopoietic stem and progenitor cells
Source: PLoS One. 2018 May 31;13(5):e0197233. doi: 10.1371/journal.pone.0197233 (PMC5979002; doi:10.1371/journal.pone.0197233)
Supplement: S2 Table — (PDF) [file pone.0197233.s004.pdf]

S2 Table. Sample information: control samples from healthy blood donors.

Sample	Sex	Age
Ctrl1	m	56
Ctrl2	m	53
Ctrl3	m	62
Ctrl4	f	61
Ctrl5	f	66
Ctrl6	m	48
Ctrl7	m	35
Ctrl8	m	25
Ctrl9	m	44
Ctrl10	m	47
Ctrl11	m	51
Ctrl12	m	71
Ctrl13	m	61
Ctrl14	m	50
Ctrl15	m	49
