# Supplementary material for: Serum of myeloproliferative neoplasms stimulates hematopoietic stem and progenitor cells
Source: PLoS One. 2018 May 31;13(5):e0197233. doi: 10.1371/journal.pone.0197233 (PMC5979002; doi:10.1371/journal.pone.0197233)
Supplement: S3 Table — (PDF) [file pone.0197233.s005.pdf]

S3 Table. Sample information: young and old healthy blood donors.

Sample	Sex	Age
O1	m	57
O2	m	53
O3	m	56
O4	w	72
O5	w	56
O6	N/A	56
O7	m	67
O8	m	51
O9	m	59
O10	m	55
O11	w	64
O12	m	57
O13	m	57
O14	m	72
O15	m	63
Y1	w	19
Y2	m	22
Y3	m	23
Y4	w	24
Y5	m	24
Y6	m	22
Y7	w	24
Y8	w	21
Y9	m	24
Y10	m	21
Y11	m	21
Y12	m	21
Y13	m	21
Y14	w	22
